# A scoping review of longitudinal studies of athlete burnout

**DOI:** 10.3389/fpsyg.2025.1502174

**Published:** 2025-02-14

**Authors:** Beate Evelīna Dišlere, Kristīne Mārtinsone, Jeļena Koļesņikova

**Affiliations:** ^1^Department of Health Psychology and Paedagogy, Faculty of Health and Sports Sciences, Riga Stradiņš University, Riga, Latvia; ^2^Department of Health Psychology and Paedagogy, Psychology Laboratory, Faculty of Health and Sports Sciences, Riga Stradiņš University, Riga, Latvia

**Keywords:** burnout, athlete, longitudinal, sport, psychology

## Abstract

**Introduction:**

Prior research has shown that increasing training and competition loads, along with associated stressors, can negatively impact athletes’ mental health and contribute to burnout. While athlete burnout can be associated with various negative sports-related consequences, such as withdrawal from sports or injuries. Although most studies on athlete burnout employ cross-sectional designs, longitudinal approaches could provide valuable insights into athlete burnout changes over time and potential causal relationships between variables and burnout. Therefore, this study aims to systematically examine longitudinal design studies to offer a comprehensive methodological, conceptual, and practical overview of athlete burnout and its associated factors.

**Methods:**

Following PRISMA-ScR guidelines, this review explores what factors influence changes in burnout levels among athletes throughout a sports season. Therefore, studies were selected that examined athlete burnout across both genders, all age groups, and various sport types, using repeated measurements. Published articles from 2014 to 2024 were collected. Eligible studies were identified through three databases: PubMed, Scopus, and Web of Science.

**Results:**

A total of 32 studies were analyzed. Quantitative mapping highlights study demographics, measurement approaches, and procedures, while qualitative mapping identifies 26 factors categorized as risk, protective, and factors influenced by burnout. The review highlights the use of tools like the Athlete Burnout Questionnaire and identifies optimal data collection intervals for tracking burnout dynamics.

**Conclusion:**

This scoping review offers insights into the multidimensional and nonlinear nature of athlete burnout, emphasizing its development through longitudinal studies and the importance of monitoring specific dimensions. The findings revealed various athlete burnout influencing personal and sport-environmental factors, including risk factors like perfectionistic concerns and negative social interaction, protective factors such as resilience-related skills and relatedness, and social support. The study emphasizes the importance of early detection and longitudinal monitoring to prevent burnout and mitigate its impact on athletes’ mental health and performance. Further research is needed to explore additional risk and protective factors to develop effective interventions aimed at reducing the risk of burnout in athletes.

## 1 Introduction

Burnout is a mental health problem that appears to be increasingly common among athletes in recent years (Madigan et al., 2022). Importantly, burnout may additionally raise the risk of acquiring both mental and physical health disorders (Glandorf et al., 2023; Wilczyńska et al., 2022). A systematic review of mental and physical health outcomes of burnout in athletes showed significant results, that athlete burnout was associated with increases in negative mental health outcomes (e.g., depression, anxiety, addictive behavior, insomnia, worry, mood, psychological distress, body image dissatisfaction) and decreases in positive mental health outcomes (e.g., satisfaction, subjective wellbeing, and quality of life). Burnout negatively affects athletes in various aspects, including reducing performance, hindering interpersonal relationships, and impairing well-being (Eklund and DeFreese, 2020). However, evidence for an association between athlete burnout and physical health outcomes was mixed (Glandorf et al., 2023).

Burnout was initially observed in caregiving professions and was defined as a psychological syndrome comprised of three symptoms—reduced professional efficacy, emotional exhaustion, and depersonalization (Maslach and Jackson, 1986). Raedeke (1997) adapted Maslach and Jackson (1986) burnout concept to the sports context and athletic experience. Reduced professional efficacy was associated with sports context and defined as a reduced sense of accomplishment, regarding athletic abilities and accomplishments. Physical fatigue, which is a consequence of training and competing, was incorporated into the definition of emotional and physical exhaustion. Depersonalization was the least adaptable to the sports context, therefore Raedeke proposed to use devaluation. For athletes, devaluation appears as a negative attitude towards sport and psychological detachment from sport. Therefore, Raedeke in 1997 established athlete burnout as a psychological syndrome composed of three dimensions: physical/emotional exhaustion, reduced sense of accomplishment, and sport devaluation.

The way that burnout changes over time has been examined in numerous studies (e.g., Amemiya and Sakairi, 2022; Shannon et al., 2022; Waleriańczyk and Stolarski, 2022). These studies demonstrate that burnout in general, as well as each of its dimensions individually, is variable and can develop or diminish over time. It is proven that that the reduced sense of accomplishment dimension tends to increase over time, this could be explained by the fact that athletes respond to the increased competition load with a greater negative evaluation of their performances and abilities (Madigan et al., 2022). Also, during the competitive season, this burnout dimension increases significantly (Madigan et al., 2022). Additionally, during the sporting season and generally, over time, the sport devaluation dimension increases (Pires and Ugrinowitsch, 2021). Athletes evaluate their athletic ability more negatively and feel a greater need to disassociate themselves from their sport (Madigan et al., 2022). The dimension of physical and emotional exhaustion shows less discernible changes over time, and it also varies less with the season (Madigan et al., 2022; Pires and Ugrinowitsch, 2021).

Several models have been proposed to explain the development of athlete burnout. The scenario in which athletes evaluate sports practice as a source of stress provides support for the cognitive-affective model (CAM) of burnout proposed by Smith (1986). According to this model, the imbalance between the demands of a situation and the coping resources to deal with them can lead to stress. As noted in a recent meta-analysis and longitudinal studies, stress is one of the factors most strongly related to athlete burnout (Lin et al., 2022; Madigan et al., 2022; Nixdorf et al., 2020; Pires and Ugrinowitsch, 2021). Another theory discussed in this context is self-determination theory (SDT; Deci and Ryan, 1985). According to SDT, the satisfaction of the core human needs of autonomy (perceptions of control and self-endorsement of an activity), competence (perceptions of proficiency), and relatedness (connection with others) are fundamental for optimal psychological well-being and human functioning (Gustafsson et al., 2017). When needs are satisfied, athletes develop intrinsic motives for participation and experience optimal health. In sports, intrinsic motivation is associated with an athlete’s interest and enjoyment that is derived from participating in sports, however, a lack of motivation is connected to an athlete’s lack of self-determination (Kinoshita et al., 2023). An additional model that explains the burnout of athletes is the sport commitment model (Schmidt and Stein, 1991), which is an etiological paradigm of burnout, in which the variable of interest is sport commitment. Authors of the sport commitment model consider burnout syndrome in athletes according to a combination of five factors—benefits, costs, satisfaction, alternatives, and investments (De Francisco et al., 2022). In 1997, Raedeke presented a commitment-based model (CBM; Raedeke, 1997), representing the desire and resolve to continue participating in sports. Commitment is perceived as the outcome of three elements:

1. How attractive or enjoyable the activity is perceived;
2. Which alternatives to the activity are viewed as in a greater or lesser degree attractive;
3. Restrictions the athlete perceives to withdraw from sport such as personal investments and social constraints.

How athletes interpret these facets determines whether their commitment is based on attraction (“want to”) or entrapment (“have to”). According to this perspective, athletes who burn out do so because they are committed solely for entrapment reasons (Madigan et al., 2022). Raedeke (1997) characterized athlete commitment as having the “two faces” of attraction and entrapment. It was believed that athletes who considered their activity to be intrinsically rewarding and who wished to participate in it were exhibiting attraction-based commitment. On the other hand, athletes were seen to be exhibiting entrapment-related commitment if they were no longer intrinsically motivated to participate in the sport and felt obligated to continue playing while no longer having an innate desire to do so (Raedeke, 1997).

These models highlight a variety of elements that contribute to athlete burnout, both individually and environmentally (in a sports context). It can be difficult to recognize and avoid a wide range of these issues. Individual and environmental issues should be addressed together for each case as part of a multifaceted therapeutic strategy. Indeed, using an individual-organization fit paradigm may prove particularly beneficial in the prevention of burnout (DeFreese et al., 2015).

Even mild signs emphasize the importance of early burnout identification, which, if neglected, can have a considerable influence on sports performance (Hassmén et al., 2023). Reducing adolescent athlete burnout could be essential for the general growth of youth in society (Wilczyńska et al., 2022). It is possible to observe changes in athlete burnout scores by conducting longitudinal studies, in which a variable or group of variables in the same cases or participants is studied over a period of time, sometimes several years (American Psychological Association, 2018). There are longitudinal studies of athlete burnout, but no review has been done where all of these studies were gathered and examined. As such, the purpose of this paper is to systematically examine longitudinal design studies to provide an overview of athlete burnout development and its associated factors.

## 2 Method

The scoping review was conducted following the Steps for Conducting a Scoping Review (Mak and Thomas, 2022), and PRISMA for Scoping Reviews guidelines (PRISMA-ScR; Tricco et al., 2018). See PRISMA-ScR checklist in Supplementary material. The protocol for this review, including information related to the search strategy, data extractions, and analysis, was registered in the Open Science Framework (OSF) database.^1^

A preliminary search of the literature was helpful to determine that research questions are not too broad or too narrow. Literature research was conducted to make sure whether a scoping review on this topic has already been conducted. When such a review had not yet been done, the available literature on this topic was examined. As the topic is currently relevant, enough research is available to conduct a scoping review of the topic.

### 2.1 Search strategy

The PCC Framework, which is advised for a scoping review, was applied to formulate the research question and eligibility criteria (Pollock et al., 2023). The following eligibility criteria were defined:

Population—male and female athletes of all ages, from both team and individual sports, were included in order to provide a comprehensive review of the topic. Excluding other representatives of the sports field, such as coaches.
Concept—factors influencing changes in athlete burnout.
Context—studies with a longitudinal design, with at least two data collection waves (throughout a sports season). Since research from the last 10 years is more likely to reflect the most current theories, methods, technologies, and practices in the field, this research included studies published from 2014 to 2024. By narrowing the focus to the last 10 years, the scoping review stays focused, relevant, and aligned with the most up-to-date understanding of the field. Peer-reviewed studies, published in any language, were included in this scoping review.

The following research question was formulated: What factors influence changes in burnout levels among athletes throughout a sports season?

The search was conducted in April 2024 using three databases. PubMed, Scopus, and Web of Science were selected due to its broad coverage of multidisciplinary research, including sports psychology, and its ability to index articles from various disciplines. Peer-reviewed journals, citation analysis, and coverage of the field’s theoretical and applied facets are all well-represented in these databases. PubMed focuses on psychological aspects of physical performance, mental health in athletes, and interventions aimed at improving well-being in sports settings. Scopus offers multidisciplinary coverage with citation analysis, helping to identify key studies and leading authors in the field of athlete burnout. Web of Science’s citation tracking allows to identify foundational publications and trace the evolution of burnout theories. Together, these databases provide a comprehensive overview of the field.

After identifying the research question and eligibility criteria, a consultation with a principal librarian about search strategy and terms was organized. Key search terms were combined using Boolean operators: (“burnout s” OR “burnout, psychological” OR (“burnout” AND “psychological”) OR “psychological burnout” OR “burnout” OR “burnouts”) AND (“athlete s” OR “athletes” OR “athlete” for athlete burnout), (“longitudinal studies” OR (“longitudinal” AND “studies”) OR (“longitudinal” AND “study”) OR “longitudinal study”).

### 2.2 Data extraction

**Figure 1:**
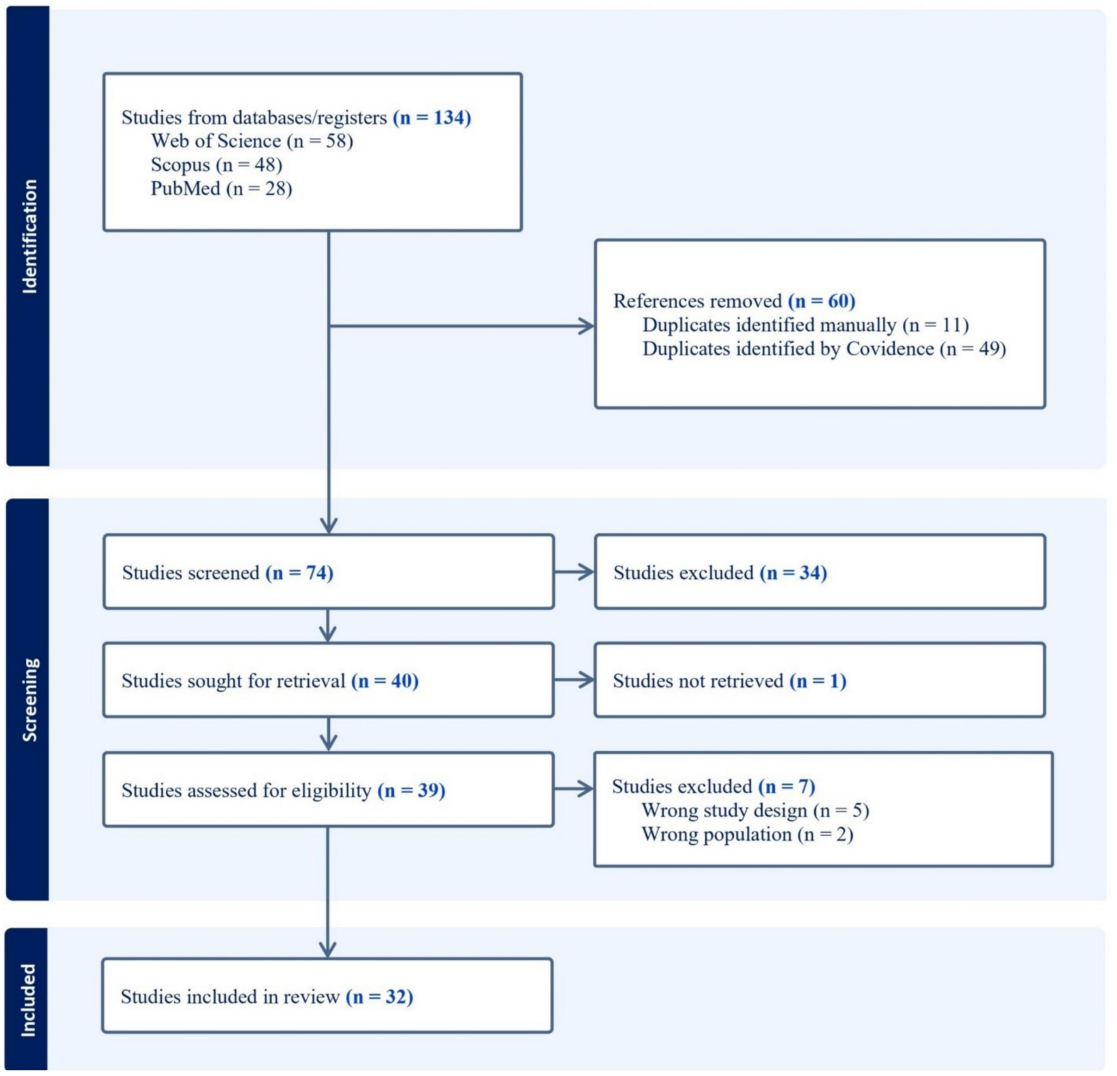
PRISMA flow diagram for information search.

Studies selected by titles and keywords (*n* = 134) were placed in the online software tool—Covidence. Covidence identified 49 duplicates, and 11 duplicates were identified manually. The first and second authors first screened titles, then abstracts, and in the final full texts. If the system showed inconsistencies about the authors’ included or excluded studies, a third author was involved and reviewed each case. The most common reason for excluding the article was that there were no repeated measurements of athletes’ burnout or the population was the wrong one (e.g., sports coaches). See Preferred Reporting Items for Systematic reviews and Meta-Analyses (PRISMA) flow diagram for an overview of information search process (Page et al., 2021) (see Figure 1).

The first author performed data extraction in the data charting form (see Table 1). The articles selected for the study (*n* = 32) were individually analyzed. Data items relevant to the review question and aim, such as—author(s), title, publication year, country, research aim, population description, sport type, research methods, measurements, data collection procedure, key findings, limitations, and future directions—were collected and summarized in a table developed by the authors.

**Table 1:** Overview of studies included in the scoping review.

Nr	Author(s)/year	n, Mage (SD, Range), %females	Sport type	Measurements	Procedure	Main findings
1	Amemiya and Sakairi (2019)	*N* = 125, Mage = 19,84 (SD = 1.04), 40%	Tennis, soft tennis, dance, football, softball, and soccer.	(1) AMQ; (2) SAS; (3) BOSA; (4) PPES	2 times (average of 3 months between surveys).	Mindfulness, mediated by alexithymic tendencies two months later, was negatively associated with burnout scores 4 months later.
2	Crowell and Madigan, 2021	*N* = 170, Mage = 19.5, 44%	Soccer, football, softball, volleyball, cross country, baseball, and in others (e.g., tennis).	(1) PCI; (2) ABQ	T1 at the beginning of the athlete’s competitive season, T2 at the end of the season.	The perfectionistic concerns cognitions dimension predicted increases in the reduced sense of accomplishment and devaluation dimensions of burnout. Perfectionistic strivings cognitions were negatively correlated with devaluation.
3	Amemiya and Sakairi (2022)	*N* = 143, Mage = 19.83 (SD = 1.03), 38%	Tennis, soft tennis, taekwondo, dance team sport, American football, soccer, softball.	(1) BOSA; (2) K-10	3 times (average of 3 months between surveys).	Cross-lagged modeling showed that both interpersonal and emotional exhaustion toward athletic practice predicted a future lack of personal accomplishment and devaluation among athletes, varying slightly depending on time. In addition, research suggested that the relationship between each burnout symptom and depression may be affected by the season.
4	DeFreese and Smith (2014)	*N* = 429, M = 19.7 (SD = 2.3, range 18–24), 59%	Swimming, track and field.	(1) SSQSR; (2) PANSE; (3) SMS; (4) PSS; (5) ABQ; (6) SWLS	3 additional seasonal time points (i.e., 27 days apart).	Study results showcase burnout as a negative temporal contributor to athlete well-being. Social support was negatively associated with global burnout and emotional/physical exhaustion but not reduced accomplishment and devaluation across a sport season. Negative social interactions positively associated with global burnout and emotional/physical exhaustion but not reduced accomplishment and devaluation across a sport season.
5	Frank et al. (2017)	*N* = 194, Mage = 15.08 (SD = 1.95), 0%	Mountain biking, badminton, gymnastics, swimming, speed skating, short track, soccer, and hockey.	(1) CES-D; (2) ABQ; (3) TICS; (4) Current state of recovery	T1 preparation; T2 competition and T3 recovery phase.	Depression and burnout were both associated with chronic stress. Therefore, burnout and depression can cause each other to some degree and no particular direction can be specifically supported by the current study.
6	Fransen et al. (2022)	*N* = 279, Mage = 20.79 (SD = 3.96, range 14–34), 54%	Basketball, volleyball, soccer, ice hockey, Nordic skiing.	(1) PeerMCYSQ; (2) CREST; (3) Well-being; (4) ABQ; (5) Performance and their team’s performance	T1 in respective regular season competition early; T2 late in respective regular season competition (average of 19 weeks between questionnaire).	Findings indicated that athletes’ team identification mediated the relationship between athlete leaders’ identity leadership and both team outcomes (i.e., task climate, team resilience, and team performance) and individual outcomes (i.e., burnout, health, and individual performance).
7	Guo et al. (2022)	*N* = 352, Mage = 18.83 (SD = 4.27), 41%	Several professional sports teams.	(1) GQ-6; (2) AEQ; (3) ABQ	2 times—1 year gap.	Athlete gratitude is a significant negative predictor of athlete burnout, and also a significant positive predictor of athlete engagement. Athlete engagement and athlete burnout are mutually causal and can be mutually predicted. Athlete gratitude indirectly affects athlete burnout through athlete engagement, and also indirectly affects athlete engagement through athlete burnout.
8	Ingrell et al. (2019)	*N* = 78, Mage = 12.7 (SD = 0.44), 38%	Soccer, ice hockey, figure skating, floorball, swimming, diving, basketball, badminton, and tennis.	(1) ABQ; (2) TEOSQ	6 times (4 months between surveys).	Task orientation was significantly and negatively related to a reduced sense of accomplishment and sport devaluation. Furthermore, by focusing on the within-person effect of achievement goals, this study provides findings that support a motivational approach to the longitudinally study of burnout propensity among young student-athletes.
9	Isoard-Gautheu et al. (2015)	*N* = 895, Mage = 15,67 (SD = 1.17; range = 13–18), 48%	Handball	ABQ	Twice a year (5 waves of measurement).	Results of multilevel growth models revealed that during adolescence, “reduced sense of accomplishment” linearly decreased and was higher for girls than boys. Moreover, “emotional/physical exhaustion” increased then decreased, and seemed to have been attenuated at time points in which athletes also had higher levels of “sport devaluation.” Finally, “sport devaluation” increased over time with higher increases for girls than boys.
10	Kelecek and Koruc (2022)	*N* = 303, Mage = 22 (SD = 5.66), 51%	Volleyball	(1) ABQ; (2) SCAT; (3) ISCCS	T1 beginning; T2 middle and T3 end of the season.	At the beginning of the season burnout positively correlated with competitive anxiety, but negatively correlated with coping strategies. While in the middle of the season, there is only positive correlation between burnout and competitive anxiety; at the end of the season there is still positive relation between burnout and competitive anxiety and addition to this, competitive anxiety showed positive correlation with coping strategies.
11	Květon et al. (2021)	*N* = 251, Mage = 16,65 (range 14–19), 49%	Swimming, tennis, volleyball, basketball, and football.	(1) Sport-MPS-2; (2) ABQ; (3) TDS; (4) Perceived performance	3 waves. The longitudinal sample that provided data after a 3-month interval. The longitudinal sample that provided data after a 1-year interval.	In the cross-sectional analyses, sequential regressions revealed that perfectionism was a significant predictor of athlete burnout and both indicators of overtraining.
12	Madigan et al. (2016a)	*N* = 141, Mage = 17,3 (SD = 0.8, range 16–19), 12%	Soccer, rugby, basketball, athletics, other sports (e.g., cycling, squash),	(1) Sport-MPS; (2) BRSQ; (3) ABQ	T1 beginning, T2 middle and T3 end of the season (each separated by 3 months).	The present findings indicate that athletes who are high in perfectionistic strivings tend to have higher levels of autonomous motivation and show lower levels of burnout, whereas athletes who are high in perfectionistic concerns tend to have higher levels of controlled motivation and show higher levels of burnout.
13	Madigan et al. (2015)	*N* = 103, Mage = 17,7 (SD = 0.8, range 16–19), 20%	Soccer, rugby, basketball, athletics, other sports (e.g., cycling, squash)	(1) Sport-MPS; (2) ABQ	Twice separated by 3 months.	Whereas perfectionistic concerns predicted increases in athlete burnout over the 3 months, perfectionistic strivings predicted decreases.
14	Martinent et al. (2014)	*N* = 145, Mage = 13.89 (SD = 2.03), 32%	Table tennis	(1) ABQ; (2) SMS	3 time points (1 month between each completion) during a 2-month period.	Results suggest that athlete burnout predicts motivation over time but motivation did not predict athlete burnout over time.
15	Martinent et al. (2020)	*N* = 159, Mage = 14.07 (SD = 2.13), 31%	Table tennis	ABQ	3 time points (1 month between each completion) during a 2-month.	3 distinct trajectories for each athlete burnout dimension, not only indicating linear or quadratic change, but also stability in longitudinal athlete burnout perceptions. Results suggested that reduced accomplishment predicted changes in the 2 other athlete burnout dimensions.
16	Scotto di Luzio et al. (2020)	*N* = 250, Mage = 15.65 (SD = 1.6), 41%	I.e. boxing, cycling, swimming, gymnastics, basketball, football, and handball.	(1) SSCAQ; (2) ABQ; (3) BRSQ; (4) UWES	T1 beginning of the season, T2 middle or end of the season (5 months apart)	Athlete burnout is associated with negative consequences for athletes’ well-being, whereas engagement reflects a positive state. In this study, we provided preliminary evidence of the protective role of the sport sense of community on athlete burnout. Consistent with our hypothesis, sport sense of community dimensions (i.e., satisfaction of needs and influence) negatively predicted athlete burnout and controlled motivation, and positively predicted engagement and autonomous motivation six months later.
17	Pires and Ugrinowitsch (2021)	*N* = 44, Mage = 25.57 (SD = 4.72, range 18–35), 59%	Volleyball	(1) ABQ; (2) ACSI-28BR	T1 in the preseason; T2 during the State Championships and/or during friendly; T3 during the first leg of the National Volleyball League; T4 during the last leg of the National Volleyball League and the Continental Club Championship.	Burnout dimensions showed a moderate inverse correlation to confidence motivation at all measurement points during the season. However, no coping differences were observed through the longitudinal analysis.
18	Santos-Afonso et al. (2023)	*N* = 64, Mage = 13.5, 05	Handball	ABQ	T1 upon arrival of the athletes at the National Handball Development Center; T2 upon departure of the athletes, after 10 days of immersion in the National Camp.	At the end of the camp, the athletes presented statistically higher means for all subscales of the ABQ and for total burnout in relation to answers obtained upon arrival at the camp.
19	Shannon et al. (2022)	*N* = 605, Mage = 24.04 (SD = 5.56), 32%	Interactive team sports and individual sports.	(1) BPNSFS; (2) ABQ	T1 baseline Week 0; T2 post 12 weeks following base-line.	Higher burnout at baseline predicted an increase in autonomy frustration, whereas higher relatedness satisfaction at baseline reduced burnout levels later in the season.
20	Smith et al. (2018)	*N* = 162, Mage = 16,15 (SD = 1.84, range 14–21), 0%	Soccer	(1) FMPS-Brief; (2) ABQ; (3) CES-D	Twice separated by 3 months.	Socially prescribed perfectionism (SPP) predicts increases in exhaustion over time. SPP predicted changes in devaluation and at the same time devaluation predicted changes in SPP. The only instance in which SPP did not predict a burnout symptom was for a sense of reduced accomplishment. Self-oriented perfectionism did not predict any burnout symptoms over time nor was it predicted by any burnout symptoms.
21	Sorkkila et al. (2020)	*N* = 391, Mage = 16 (SD = 0.17, range 15–16), 51%	Team sports and individual sports.	SBI	T1 at the beginning of the first semester; T2 6 months later at the end of the school year.	Four different burnout profiles were identified. Those elite athletes who are at risk of burnout might suffer particularly from school burnout symptoms, which then spill over into the sport context. The athletes in the Burnout risk profile showed a relatively high level of sport and school burnout symptoms at the beginning of upper secondary school, and the level of their school burnout symptoms remained relatively steady over 6 months. However, the level of the sport burnout symptoms in this group decreased over time. In the Developed burnout group, the athletes initially showed few sport and school burnout symptoms, although both kinds of symptoms increased significantly over a 6-month period.
22	Sorkkila et al. (2019)	*N* = 391, Mage = 16 (SD = 0.17), 51%	Team sports (e.g., football, ice hockey) and individual sports (e.g., gymnastics, skiing).	(1) SBI; (2) BRS; (3) Sport dropout	T1 at the beginning of their first year in upper secondary sport school; T2 6-months later at the end of the first school year; T3 1 year later at the end of the second school year; T4 finally, 6 months later in the beginning of the third school year.	In the increased burnout group symptoms were less resilient and more likely to dropout from sport than those in the other two groups. Athletes with increased burnout are less resilient and more likely to dropout from sport.
23	Zhang et al. (2023)	*N* = 515, Mage = 18.24 (SD = 3.16, range 12–31), 44%	No information.	(1) AMQ; (2) AAQ-II; (3) CFQ; (4) ABQ	Three times.	Findings provide insights on the changing mechanisms how mindfulness can reduce athlete burnout. That is, athletes with higher levels of mindfulness have low levels of burnout due to their low levels of experiential avoidance and cognitive fusion. These findings provided evidence supporting the working mechanism that athlete with higher tendencies towards the avoidance of unpleasant private experiences and high levels of fusions with the painful thoughts and feelings normally have high levels of burnout.
24	Madigan and Nicholls (2017)	*N* = 102, Mage = 17.7 years (SD = 0.7; range 16 to 20), 27,5%	Soccer, rugby, basketball, athletics or other sports.	(1) MTI; (2) ABQ	2 times with 3-month interval	Mental toughness predicted residual decreases in total burnout, reduced sense of accomplishment, physical and emotional exhaustion, and devaluation over time. Mental toughness predicted residual decreases in burnout over time.
25	Madigan et al. (2016b)	N = 129, Mage = 24.8 (SD ¼ 5.1; range ¼ 20 to 35 years), 48,3%	Athletics, netball, gymnastics, rugby, cycling, soccer, and other sports (e.g., basketball, cricket).	(1) Sport-MPS; (2) ABQ	2 times with 3-month interval	When moderated regression analyses were employed, interactive effects of evaluative concerns perfectionism × personal standards perfectionism were found indicating that personal standards perfectionism buffered the effects of evaluative concerns perfectionism on total burnout and physical/emotional exhaustion.
26	Pires et al. (2016)	N = 15, Mage = 24,00 (SD = 3,55), 0%	Volleyball	(1) ABQ; (2) ACSI-28	T1 pre-season, T2 State Championship, T3 main competition in country, T4 in play-offs’.	The results show an increase in the perception of burnout in the athletes with the build-up of training and competition, as well as the requirement for optimal performance in the main competition. The study also showed the importance of the motivational factor in the emergence of burnout.
27	Ueno and Suzuki (2016)	N = 63; Mage = 19.4 (SD = 1.1), 62%	Softball, baseball, track and field events, badminton, and lacrosse	(1) RSUA; (2) BOSA	3 times (average of 3 months between surveys).	The results of this study suggest a new perspective on the resilience competency of athletes to recovery their mental health. Applying the concept of resilience to sports may prevent burnout and the dropout rate of athletes, and it may maintain and improve their mental health
28	Waleriańczyk and Stolarski (2022)	N = 173, Mage = 27.47 (SD = 6.11, range 18 to 39), 49%	Short and long distance running, football, combat sports, archery, basketball, and tennis.	(1) SMPS2; (2) PPS-S; (3) ABQ; (4) SES	2 times, 5 months apart.	We provide further support for the notion that when perfectionism—burnout link is studied longitudinally personal standards perfectionism shows burnout-decreasing effects, while evaluative concerns perfectionism is maladaptive at least for exhaustion and reduced sense of accomplishment. We also report pioneering evidence that perfectionism predicts changes in athlete engagement, with evaluative concerns perfectionism showing maladaptive effects for all the dimensions and personal standards perfectionism showing engagement-increasing effects limited to the dedication subscale only.
29	Gerber et al. (2018)	N = 257; Mage = 16.8 (SD = 1.4), 37%	Team sports and individual sports.	(1) SMBM; (2) ISI; (3) Sleep-EEG assessments	2 times, 6 months apart.	In the present study, between 12 and 14% of young elite athletes reported clinically relevant burnout symptoms, whereas 4–11% reported clinically relevant insomnia symptoms. Athletes with clinically relevant burnout were more likely to report insomnia symptoms. Moreover, baseline burnout symptoms predicted increased insomnia symptoms over time.
30	Li et al. (2018)	N = 10, Mage = 24.80 years (SD = 2.53), 0%	Blind soccer	(1) ABQ; (2) PSQI	6 times, 1 month apart.	This five-wave longitudinal survey demonstrated that burnout and sleep are not reciprocally related in blind elite soccer players. The results in the present study suggest that burnout may be a risk factor of sleep problems among athletes but not vice versa.
31	Mellano et al. (2022)	N = 126, Mage = 19.78, (SD = 1.28, range 18 to 22), 100%	Field hockey, swimming and diving, basketball, track and field, softball, and lacrosse.	(1) ABQ; (2) SCQ; (3) CFQ; (4) Perceived seasonal success	T1 within the first 2 to 3 weeks of the season, T2 during the last part of the season just before playoffs.	The results of the current study indicate burnout levels in collegiate athletes may change from early to late season. Furthermore, significant predictors of late season burnout levels include early season burnout scores, coaches’ use of an autonomy-supportive interpersonal style, coaches’ use of punitive feedback, and athletes’ perceptions of low personal accomplishment.
32	Glandorf et al. (2024)	N = 267, Mage = 20.87, (SD = 4.69), 44%	Team sports and individual sports.	(1) ABQ; (2) PSI; (3) WURSS-11; (4) CESD; (5) PSQI; (6) SWLS	T1 beginning, T2 middle and T3 end of the season (6 month period).	At the between-person level, we found athlete burnout to be associated with all examined health variables. At the within-person level, emotional and physical exhaustion was found to predict increases in depressive symptoms, sleep disruptions were found to predict increases in devaluation, and life satisfaction was found to predict decreases in total burnout, exhaustion, and reduced sense of accomplishment.

AAQ-II, Acceptance and Action Questionnaire – version 2; ABQ, Athlete Burnout Questionnaire; ACSI-28BR, Athletic Coping Skills Inventory-28; AEQ, Athlete Engagement Questionnaire; AMQ, Athlete Mindfulness Questionnaire; BOSA, The Burnout Scale for University Athletes; BPNSFS, 8-item Need Satisfaction and Frustration Scale; BRS, Brief Resilience Scale; BRSQ, Behavioral Regulation Questionnaire in Sport; CES-D, Center for Epidemiologic Studies Depression Scale; CFQ, Coaching Feedback Questionnaire; CFQ, Cognitive Fusion Questionnaire; CREST, Characteristics of Resilience in Sports Teams; FMPS-Brief, Multidimensional Perfectionism Scale brief version; GQ-6, Gratitude Questionnaire; ISCCS, Inventory of coping strategies in competitive sports; ISI, Insomnia Severity Index; K-10, Kessler Psychological Distress Scale; MIPS, Multidimensional Inventory of Perfectionism in Sport; MTI, The Mental Toughness Index; PANSE, Positive and Negative Social Exchanges; PCI, Perfectionistic Cognitions Inventory; PeerMCYSQ, Peer Motivational Climate in Youth Sport Questionnaire; PPES, The Psychological Performance Efficacy Scale; PSI, 18-item Physical Symptom Checklist; PPS-S, Performance Perfectionism Scale–Sport; PSQI, Pittsburgh Sleep Quality Index; PSS, Perceived Stress Scale; RSUA, Psychological Resilience Scale for University Athletes; SBI, Sport Burnout Inventory; SAS, The Sport Alexithymia Scale; SCAT, The Sport Competition Anxiety Test; SCQ, Sport Climate Questionnaire; SES, Sport Engagement Scale; SMBM, Shirom-Melamed Burnout Measure; SMPS2, Sport-Multidimensional Perfectionism Scale-2; SMS, Sport Motivation Scale; Sport-MPS, Sport Multidimensional Perfectionism Scale; SSCAQ, Sport Sense of Community in Adolescence Questionnaire; SSQSR, Social Support Questionnaire short form; SWLS, Satisfaction With Life Scale; TDS, Training Distress Scale; TEOSQ, The Task and Ego Orientation in Sports Questionnaire; TICS, Trier Inventory for Chronic Stress; UWES, Utrecht Work Engagement Scale;WURSS-11, 11-item Wisconsin Upper Respiratory Symptom Survey.

### 2.3 Data analysis

The findings from the numerical analysis are presented in a table to highlight the most salient aspects of the review. Thematic analysis was conducted based on the research purpose and questions. A list of tentative codes was created to group the information into categories. For example, all studies that examined burnout during the competitive season were combined into one category, or all studies that examined the relationship between perfectionism and burnout into another. Afterward, the results were analyzed quantitatively, summarizing the entire study demographics data, measurement approach, and procedure. Qualitative analysis and division of factors affecting athlete burnout into subsections are also needed.

## 3 Results

In total, 32 articles met the inclusion criteria (see Figure 1).

### 3.1 Quantitative mapping—characteristics of sources

#### 3.1.1 Demographics analysis

Less than half of the articles (*n* = 15) were published between 2014 and 2018, and the rest of the articles (*n* = 18) between 2019 and 2024. The most articles (*n* = 7) were conducted in 2022. Studies have been published in different countries—United Kingdom (*n* = 8), France (*n* = 4), United States of America (*n* = 2), Japan (*n* = 3), Brazil (*n* = 3), China (*n* = 3), Finland (*n* = 2), Germany (*n* = 2), Canada (*n* = 1), Czech Republic (*n* = 1), Sweden (*n* = 1), Turkey (*n* = 1), Poland (*n* = 1), Switzerland (*n* = 1). Considering demographics, the average sample size was 228 (ranging from 10 to 895). Females made up 36% of the participants, mean average age was 18.9 years. The athletes were engaged in sports for an average of 8.15 years. They were training for an average of 12.32 h per week. 70% were representatives of team sports (ice hockey, soccer, blind soccer, rugby, handball, netball, baseball, lacrosse, volleyball, basketball, American football, softball, badminton). The remaining 30% were individual athletes (running, gymnastics, running, mountain biking, cycling, short track, swimming, table tennis, skiing, track and field, tennis, taekwondo, boxing, and dance team sport). See Table 2 for study and sample characteristics.

**Table 2:** Study and sample characteristics.

Study characteristics	Reference numbers	*n*	%
Published			
2014–2016	4; 9; 12; 13; 14; 25; 26; 27	8	25
2017–2019	1; 5; 8; 20; 22; 24; 29; 30	8	25
2020–2022	2; 3; 6; 7; 10; 11; 15; 16; 17; 19; 21; 28; 31	13	41
2023–2024	18; 23; 32	3	9
Location			
Europe	2; 5; 8; 9; 11–16; 19–22; 24; 25; 28; 29; 32	19	60
North America	4; 6; 31	3	9
South America	17; 18; 26	3	9
East Asia	1; 3; 7; 23; 27; 30	6	19
Middle East	10	1	3
Sample size			
≤ 50	17; 26; 30	3	9
51–100	8; 18; 27	3	9
101–150	1; 3; 12; 13; 14; 24; 25; 31	8	24
151–200	2; 5; 15; 20; 28	5	16
201–300	6; 11; 16; 29; 32	5	16
301–400	7; 10; 21; 22	4	13
>400	4; 9; 19; 23	4	13
Gender			
Female	31	1	3
Male	5; 18; 20; 26; 30	5	16
Combined	1–4; 6–17; 19; 21–25; 27–29; 32	26	81
Age (mean)			
12–13	8; 14; 18	3	9
14–15	5; 9; 15; 16	4	13
16–17	11; 12; 13; 20; 21; 22; 24; 29	8	24
18–19	1–4; 7; 23; 27; 31; 32	9	29
20–24	6; 10; 19; 25; 26; 30	6	19
25–27	17; 28	2	6
Type of sport			
Team	7; 9; 10; 17; 18; 20; 26; 30; 31	9	29
Individual	4; 14; 15	3	9
Combined	1; 2; 3; 5; 6; 8; 11; 12; 13; 16; 19; 21–25; 27; 28; 29; 32	20	62

Percentages (%) in relation to the total number of included empirical articles (N = 32). Reference numbers: 1. Amemiya and Sakairi (2019); 2. Crowell and Madigan (2021); 3. Amemiya and Sakairi (2022); 4. DeFreese and Smith (2014); 5. Frank et al. (2017); 6. Fransen et al. (2022); 7. Guo et al. (2022); 8. Ingrell et al. (2019); 9. Isoard-Gautheu et al. (2015); 10. Kelecek and Koruc (2022); 11. Květon et al. (2021); 12. Madigan et al. (2016a); 13. Madigan et al. (2015); 14. Martinent et al. (2014); 15. Martinent et al. (2020); 16. Scotto di Luzio et al. (2020); 17. Pires and Ugrinowitsch (2021); 18. Santos-Afonso et al. (2023); 19. Shannon et al. (2022); 20. Smith et al. (2018); 21. Sorkkila et al. (2020); 22. Sorkkila et al. (2019); 23. Zhang et al. (2023); 24. Madigan and Nicholls (2017); 25. Madigan et al. (2016b); 26. Pires et al. (2016); 27. Ueno and Suzuki (2016); 28. Waleriańczyk and Stolarski (2022); 29. Gerber et al. (2018); 30. Li et al. (2018); 31. Mellano et al. (2022); 32. Glandorf et al. (2024).

#### 3.1.2 Measurement analysis

Information on the instruments used to measure athlete burnout was collected and summarized in Table 3.

**Table 3:** Measure and procedure analysis.

Instruments	Waves of data collection							
	Two competitive season points	Three competitive season points	One month apart	Three-months apart	Four months apart	Five months apart	Six months apart	One year apart
ABQ	2; 6; 16; 31	4; 5; 10; 12; 17; 18; 23; 26; 32	14; 15; 30	11; 13; 19; 20; 24; 25	8	28	9	7; 11
BOSA				1; 3; 27				
SpBI-DC							21; 22	22
SMBM							29	

ABQ, Athlete Burnout Questionnaire; BOSA, Burnout Scale for University Athletes; SpBI-DC, Sport Burnout Inventory—Dual Career Form; SMBM, Shirom-Melamed Burnout Measure.

81% of studies used the Athlete Burnout Questionnaire (ABQ; Raedeke and Smith, 2001). The ABQ consists of 15 questions, structured on a Likert scale of five points, five questions to measure the sub-areas “physical and emotional exhaustion,” five to measure the sub-area “sport devaluation,” and five to measure the sub-area “reduced sense of sports accomplishment” (Santos-Afonso et al., 2023).

The Burnout Scale for University Athletes (BOSA; Amemiya et al., 2013) was used in 10% of studies. This scale is composed of 20 items, with five items from each of the following four sub-scales: “interpersonal emotional exhaustion,” “lack of personal accomplishment,” “emotional exhaustion for athletic practicing,” and “devaluation of club activities” (Amemiya and Sakairi, 2022).

6% of studies used the Sport Burnout Inventory—Dual Career Form (SpBI-DC; Sorkkila et al., 2017). The SpBI-DC is a modified version of the School Burnout Inventory (SBI; Salmela-Aro et al., 2009) and it has been developed to investigate sports burnout among student-athletes. The scale consists of 10 items, out of which four measure sport-related exhaustion, three measure cynicism toward the meaning of one’s sport, and three measure feelings of inadequacy as an athlete. All items were rated on a 5-point Likert scale (Sorkkila et al., 2019).

3% of studies or one study used the 14-item Shirom-Melamed Burnout Measure (SMBM; Lerman et al., 1999). The SMBM comprises three subscales—physical fatigue, cognitive weariness, and emotional exhaustion (Gerber et al., 2018).

#### 3.1.3 Procedure analysis

Since only longitudinal studies were collected, an analysis of the procedure of the studies, comparing the frequency and waves of data collection was also performed. See Table 3.

The most frequently used data collection method was at three competitive season points (T1 = preparation phase, T2 = competition phase, and T3 = recovery phase), used in 9 studies (26.5%).

A comparable way of collecting data was at two competitive season points (T1 = within the first 2 to 3 weeks of the season, T2 = during the last part of the season just before playoffs), used in 4 studies (12%). The average interval between each completion was 5 months.

The second most frequently used method of data collection was to collect data separated by a three-month period, and this method was used in 9 studies (26.5%). The three-month interval between waves was considered sufficient because previous research has shown that this time interval allows researchers to capture changes in athlete burnout during periods of active training (e.g., Cresswell and Eklund, 2005; Madigan et al., 2016a; Madigan and Nicholls, 2017).

The other studies (35%) had different data collection intervals—6 months (*n* = 4), 1 month (*n* = 3), 1 year (*n* = 3), 4 months (*n* = 1), and 5 months (n = 1).

### 3.2 Qualitative mapping

#### 3.2.1 Athletes’ burnout related factors

**Figure 2:**
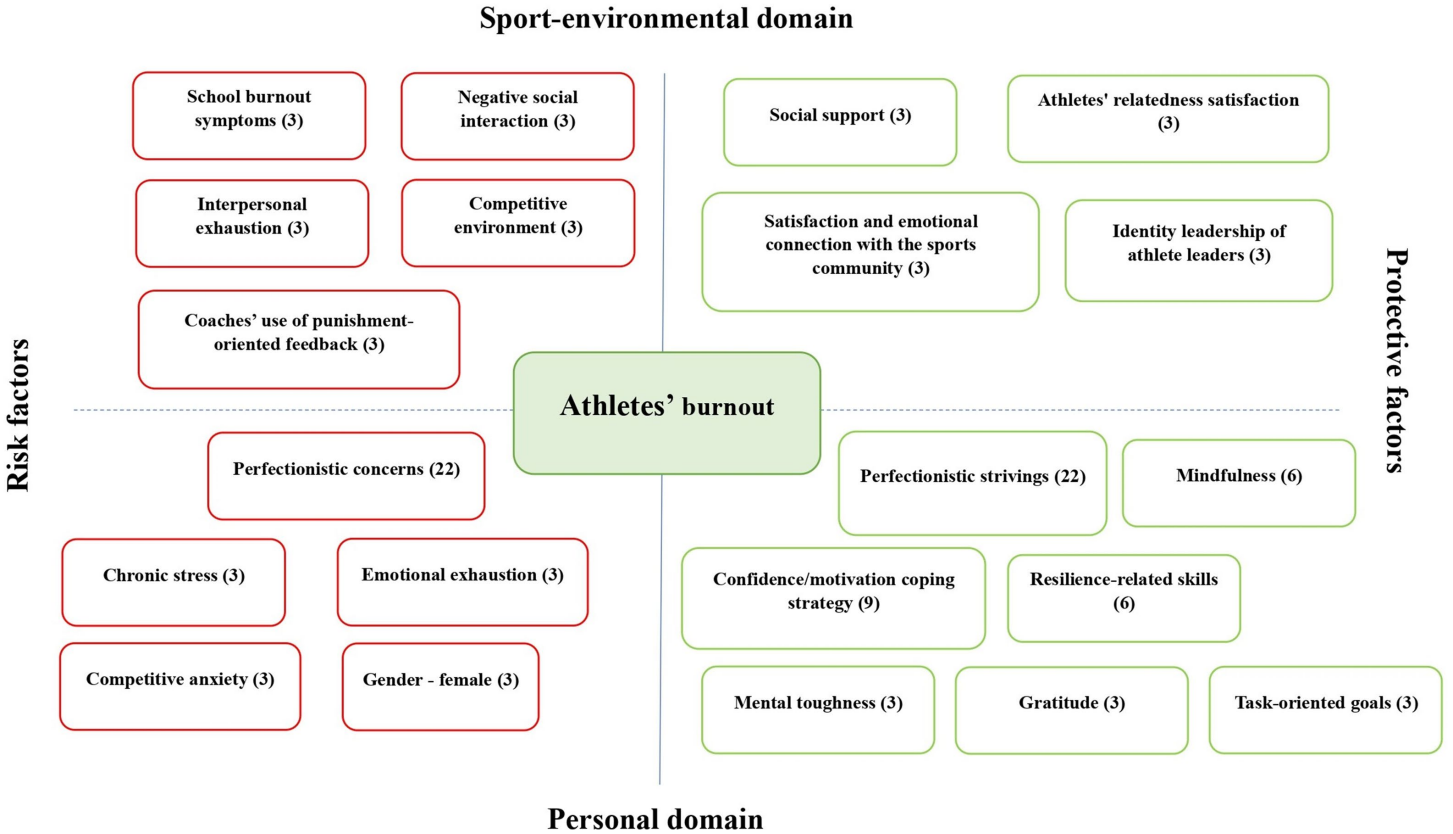
Conceptual map of protective and risk factors. Numbers in brackets indicate percentages in relation to the total number of included empirical articles (*N* = 32).

In order to answer the last research question, this chapter summarizes all the athlete burnout-related factors mentioned in the selected studies. These factors were grouped into 26 overarching themes during the analysis and were further divided into several sections—protective factors (*n* = 12), risk factors (*n* = 11) and affected by burnout factors (*n* = 3) (see Table 4). Protective and risk factors were further analyzed and divided into two subsections—personal and sport-environmental factors (see Figure 2).

**Table 4:** Personal and sport-environmental protective and risk factors affecting athletes’ burnout.

Factor themes	Reference numbers	*n*	%
Personal protective factors			
Confidence/motivation coping strategy	10; 26; 17	3	9
Mindfulness	1; 23	2	6
Perfectionistic strivings	2; 11; 12; 13; 20; 25; 28	7	22
Task-oriented goals	8	1	3
Gratitude	7	1	3
Mental toughness	24	1	3
Resilience-related skills	22; 27	2	6
Life satisfaction	32	1	3
Sport-environmental protective factors			
Social support	4	1	3
Athletes’ relatedness satisfaction	19	1	3
Identity leadership of athlete leaders	6	1	3
Satisfaction and emotional connection with the sports community	16	1	3
Personal risk factors			
Perfectionistic concerns	2; 11; 12; 13; 20; 25; 28	7	22
Emotional exhaustion	3	1	3
Gender—female	9	1	3
Competitive anxiety	10	1	3
Chronic stress	5	1	3
Sleep disruptions	32	1	3
Sport-environmental risk factors			
Coaches’ use of punishment-oriented feedback	31	1	3
Competitive environment	18	1	3
Negative social interaction	4	1	3
School burnout symptoms	21	1	3
Interpersonal exhaustion	3	1	3
Affected by burnout			
Motivation	14	1	3
Sleep	29; 30	2	6
Depressive symptoms	32	1	3

Percentages in relation to the total number of included empirical articles (N = 32). Reference numbers: 1. Amemiya and Sakairi (2019); 2. Crowell and Madigan (2021); 3. Amemiya and Sakairi (2022); 4. DeFreese and Smith (2014); 5. Frank et al. (2017); 6. Fransen et al. (2022); 7. Guo et al. (2022); 8. Ingrell et al. (2019); 9. Isoard-Gautheu et al. (2015); 10. Kelecek and Koruc (2022); 11. Květon et al. (2021); 12. Madigan et al. (2016a); 13. Madigan et al. (2015); 14. Martinent et al. (2014); 16. Scotto di Luzio et al. (2020); 17. Pires and Ugrinowitsch (2021); 18. Santos-Afonso et al. (2023); 19. Shannon et al. (2022); 20. Smith et al. (2018); 21. Sorkkila et al. (2020); 22. Sorkkila et al. (2019); 23. Zhang et al. (2023); 24. Madigan and Nicholls (2017); 25. Madigan et al. (2016b); 26. Pires et al. (2016); 27. Ueno and Suzuki (2016); 28. Waleriańczyk and Stolarski (2022); 29. Gerber et al. (2018); 30. Li et al. (2018); 31. Mellano et al. (2022); 32. Glandorf et al. (2024).

#### 3.2.2 Personal protective factors

This section summarizes information on eight different protective factors for athlete burnout.

The most frequently studied predictor of athlete burnout in longitudinal studies was perfectionism. Seven studies (Crowell and Madigan, 2021; Květon et al., 2021; Madigan et al., 2015, 2016a, 2016b; Smith et al., 2018; Waleriańczyk and Stolarski, 2022) aimed to examine the relationship between perfectionism and athlete burnout. Longitudinal results showed that *perfectionistic strivings* played a significant role in predicting change in burnout in the “long-term” (one-year) perspective, compared to the “short-term” (three-months) longitudinal results (Květon et al., 2021). Also, Madigan and colleagues studied changes over a three-month period and concluded that perfectionistic strivings predicted decreases in athlete burnout over time (Madigan et al., 2015, 2016a). At the level of burnout dimensions, it was discovered that perfectionistic striving cognitions were negatively correlated with devaluation (Crowell and Madigan, 2021; Waleriańczyk and Stolarski, 2022). Three studies investigated *coping strategies* through the longitudinal. Kelecek and Koruc (2022) study obtained the result that at the beginning of the season burnout negatively correlated with coping strategies, however, two other studies proved that confidence/motivation coping strategy has a negative and moderate correlation between the reduced sense of athletic accomplishment burnout dimension at all four times of the season (Pires et al., 2016; Pires and Ugrinowitsch, 2021). Two studies (Amemiya and Sakairi, 2019; Zhang et al., 2023) revealed that *mindfulness* has an indirect effect on athlete’s burnout scores. The relationship between *resilience* and burnout was investigated in two studies (Sorkkila et al., 2019; Ueno and Suzuki, 2016), where it was endorsed that resilience in sports may prevent burnout and the dropout rate of athletes, and it may maintain and improve their mental health. Further three more protective factors will be listed, and each one of them was investigated in only one study. Madigan and Nicholls (2017) longitudinally examined the relationship between *mental toughness* and burnout and the results showed a significant negative cross-sectional association. Guo et al. (2022) proved that athlete *gratitude* indirectly affects athlete burnout through athlete engagement and indirectly affects athlete engagement through athlete burnout. Ingrell et al. (2019) examined the within-person relationship between achievement goals and burnout perceptions. It was concluded that *task orientation* (where success is based on self-referenced criteria) was significantly and negatively related to a reduced sense of accomplishment and sport devaluation. However, no significant relationship was found between task orientation and emotional and physical exhaustion. No significant within-person relationships were found between ego orientation (where success is based on normative standards) and the three burnout variables. Glandorf et al. (2024) examined relationships between athlete burnout and several health variables. Results suggest that *life satisfaction* predicted decreases in total burnout, exhaustion, and a reduced sense of accomplishment at the within-person level. This means that high life satisfaction can be a significant resource for the athlete in reducing the symptoms of burnout.

#### 3.2.3 Sport-environmental protective factors

Four different sport-environmental protective factors were investigated in one study each. DeFreese and Smiths’ (2014) study results provide preliminary longitudinal evidence that *social perceptions* are important to athletes’ psychological health and highlight the negative contribution of burnout perceptions to athletes’ life satisfaction. Scotto di Luzio et al. (2020) study results showed that athletes who were *satisfied and emotionally connected in the sports community* had lower scores of burnout. Fransen et al. (2022) study findings indicated that the *identity leadership* of athlete leaders was positively related to teammates’ well-being and negatively to their burnout, while the same relationships for coaches’ identity leadership were not significant. Shannon et al. (2022) longitudinal associations found evidence that higher levels of *relatedness satisfaction* at the beginning of a season related to lower levels of burnout later in the season and offered a protective effect.

#### 3.2.4 Personal risk factors

Seven studies highlighted the negative effect on the athlete burnout (Crowell and Madigan, 2021; Květon et al., 2021; Madigan et al., 2015, 2016a, 2016b; Smith et al., 2018; Waleriańczyk and Stolarski, 2022). Longitudinal results showed that *perfectionistic concerns* predicted increases in athlete burnout over a three-month period of time (Madigan et al., 2015, 2016a). At the level of burnout dimensions, it was discovered that the perfectionistic concerns cognitions dimension predicted increases in the reduced sense of accomplishment and devaluation dimensions of burnout (Crowell and Madigan, 2021; Waleriańczyk and Stolarski, 2022). Smith et al. (2018) study concluded that socially prescribed perfectionism (which is considered an indicator of perfectionistic concerns) predicted changes in devaluation and devaluation predicted changes in socially prescribed perfectionism. However, socially prescribed perfectionism did not predict a burnout symptom for a sense of reduced accomplishment. Furthermore, Amemiya and Sakairi’s (2022) cross-lagged modeling showed that *emotional exhaustion* toward athletic practice predicted a future lack of personal accomplishment and devaluation among athletes, varying slightly depending on time. Frank et al. (2017) gathered information on burnout and depression, and after multiple linear regression analyses concluded that depression and burnout were both associated with *chronic stress*. Stress was a significantly better predictor for both burnout and depression than each was for the other. Isoard-Gautheu et al. (2015) study results of multilevel growth models revealed that during adolescence, reduced sense of accomplishment linearly decreased and was higher for *girls* than boys. Moreover, emotional/physical exhaustion increased then decreased, and seemed to have been attenuated at time points in which athletes also had higher levels of sport devaluation. Finally, sport devaluation increased over time with higher increases for girls than boys. Kelecek and Koruc (2022) study concluded that at the beginning of the season burnout positively correlated with *competitive anxiety*, but negatively correlated with coping strategies. In the middle of the season, there was only a positive correlation between burnout and competitive anxiety, and at the end of the season, there was a positive relationship between burnout and competitive anxiety. Studies on the effect of sleep on burnout in athletes have mixed results. In one of the studies, it was proven that sleep disruptions predicted increases in sports devaluation at the within-person level, so it can be added to one of the risk factors (Glandorf et al., 2024).

#### 3.2.5 Sport-environmental risk factors

Five different sport-environmental risk factors were investigated in one study each. Athlete burnout can be influenced by interpersonal relationships and Mellano et al. (2022) have demonstrated a relationship between burnout and *athletes’ perception of their coaches’ use of an autonomy-supportive style.* The current study showed that all three dimensions of late-season burnout were significantly and negatively related to athletes’ perception of their coaches’ use of an autonomy-supportive style. Continuing on interpersonal relationships, DeFreese and Smith (2014) provided that study results provide preliminary longitudinal evidence that *social perceptions* are important to athletes’ psychological health and highlight the negative contribution of burnout perceptions to athletes’ life satisfaction. Negative social interactions were positively associated with global burnout and emotional/physical exhaustion but not with reduced accomplishment and devaluation across a sports season. Amemiya and Sakairi’s (2022) study proved that *interpersonal exhaustion* predicted a future lack of personal accomplishment and devaluation among athletes. Interpersonal exhaustion was the main burnout symptom that predicted future depressive symptoms among athletes. *The competitive environment* also is one of the risk factors for athlete burnout. Santos-Afonso et al. (2023) studied the experience of athletes before and after being in the National Camp for Development and Improvement of Handball Technique. The evidence shows that at the end of the camp, the athletes presented statistically higher means for all subscales of athlete burnout. Sorkkila et al. (2020) examined the development of *school and sport burnout*, therefore it was concluded that those elite athletes who are at risk of burnout might suffer particularly from school burnout symptoms, which then spill over into the sport context. The athletes in the Burnout risk profile showed a relatively high level of sport and school burnout symptoms at the beginning of upper secondary school, and the level of their school burnout symptoms remained relatively steady over 6 months. However, the level of sports burnout symptoms in this group decreased over time. In the developed burnout group, the athletes initially showed few sport and school burnout symptoms, although both kinds of symptoms increased significantly over a 6-month period.

#### 3.2.6 Affected by burnout factors

Some of the included studies produced results that are reflected in this study as factors that may be influenced by athlete burnout. A total of three studies looked at the relationship between athlete burnout and sleep problems. Two of these studies investigated that athletes with clinically relevant burnout were more likely to report *insomnia* symptoms (Gerber et al., 2018; Li et al., 2018). Moreover, baseline burnout symptoms predicted increased insomnia symptoms over time (Gerber et al., 2018). From these two studies, it can be concluded that athletes who already have some burnout symptoms are more likely to experience insomnia symptoms. Another factor that can be affected by athlete burnout is *motivation*. Study results suggest that athlete burnout predicts motivation over time, but motivation did not predict athlete burnout over time (Martinent et al., 2014). Glandorf et al. (2024) confirm the idea that burnout may be a developmental antecedent of *depression*. The results of their research showed that exhaustion predicted increases in depressive symptoms.

## 4 Discussion

The current scoping review aimed to systematically examine longitudinal design studies to provide a methodological, conceptual, and applied overview of athlete burnout development and its associated factors. Specifically, the review provides an overview of 32 studies that examined athlete burnout in longitudinal design studies and contributing factors. The review gives a summary of study characteristics, measures and procedure analysis, athlete burnout change analysis, and an overview of factors related to athlete burnout. In total, 26 factors that are related to athlete burnout were identified and categorized into three categories—risk factors, protective factors, and factors that are affected by athlete burnout. There are also two subcategories for risk and protective factors—personal protective factors (e.g., perfectionistic strivings), sport-environmental protective factors (e.g., satisfaction and emotional connection with the sports community), personal risk factors (e.g., emotional exhaustion), and sport-environmental risk factors (e.g., coaches’ use of an autonomy-supportive style).

### 4.1 Exploring athlete burnout: insights from CAM, SDT, and CBM models

The Cognitive-Affective Model (CAM), Self-Determination Theory (SDT), and Commitment-Based Model (CBM) collectively provide a comprehensive lens through which burnout can be understood. For instance, CAM emphasizes the imbalance between a situation’s demands and the coping resources to deal with them, which can lead to stress. In this case, the athlete may experience demands from themselves and others involved in sports. Several studies proved that perfectionistic concerns (e.g., concerns over making mistakes) are significant predictors of the development of athlete burnout. Coaches and those involved in the organization must be careful when collaborating with athletes, especially when encouraging them to strive for excellence. Potential maladaptive manifestations of concerns, fears, and unrealistic expectations from the social environment must be closely observed (Květon et al., 2021). From the perspective of the sports environment, the imbalance can lead to emotional exhaustion, stress, and competitive anxiety (Amemiya and Sakairi, 2022; Frank et al., 2017; Kelecek and Koruc, 2022). All these factors are related to athlete burnout and its increase. These results support the CAM model’s claim that burnout is mostly predicted by stress and ineffective coping strategies, therefore suggesting specific stress management interventions. For example, mindfulness can significantly reduce stress (Amemiya and Sakairi, 2019; Zhang et al., 2023), additionally, confidence/motivation coping techniques can be used (Pires and Ugrinowitsch, 2021; Pires et al., 2016). Athletes engaged in task-oriented goals with a focus on self-referenced criteria might help to gain control regarding their sports involvement and mitigate the focus on normative standards (Ingrell et al., 2019). Resilience is another key factor in preventing burnout. Studies suggest that athletes equipped with resilience-related skills better cope with setbacks and are less likely to drop out of sports due to burnout (Sorkkila et al., 2019; Ueno and Suzuki, 2016). In tandem, mental toughness has emerged as a vital predictor of reduced burnout symptoms, including emotional and physical exhaustion (Madigan and Nicholls, 2017). Other techniques that may influence athlete burnout were also investigated in other studies, such as gratitude, which indirectly affects athlete burnout through athlete engagement (Crowell and Madigan, 2021).

According to SDT, the satisfaction of the core human needs of autonomy, competence, and relatedness are fundamental for optimal psychological well-being and human functioning (Gustafsson et al., 2017). Therefore, athletes’ perception of their coaches’ use of an autonomy-supportive style is one of the risk factors. Athletes who perceive their coaches as supportive in fostering independence and emotional well-being are less likely to experience burnout. In addition, coaches’ use of punishment-oriented feedback was found to be positively related to athletes’ late-season levels of physical and emotional exhaustion (Mellano et al., 2022). Coach and his coaching behavior also play a significant role in this athlete burnout context. It was demonstrated that coaches’ behaviors can be a predictor of athletes’ burnout levels in all three of the subdimensions (Mellano et al., 2022). Coaches can also influence the competitive environment. For example, during a competitive season, coaches can establish an atmosphere that supports an athlete’s sense of independence and choice (Mellano et al., 2022). When pushing athletes to pursue excellence, others must use extreme caution to avoid creating maladaptive expressions of worries, fears, and irrational expectations from the social environment. The athlete’s need for connection and belonging is an essential element according to SDT. Negative social interactions (characterized by unwanted, intrusive, unhelpful, unsympathetic or insensitive, or rejecting or neglecting behaviors) fall under interpersonal relationships, a key factor in athlete burnout that is associated with emotional and physical exhaustion (DeFreese and Smith, 2014). Specific interpersonal exhaustion is one of the main burnout symptoms. Relatedness satisfaction is important for athletes, and higher levels of athletes’ relatedness satisfaction at the beginning of a season are related to a lower level of burnout later in the season (Shannon et al., 2022). Moreover, social support is crucial because it helps to satisfy the need for connection and is negatively associated with athlete burnout and emotional/physical exhaustion (DeFreese and Smith, 2014). Thus, athletes who receive greater social support and experience a higher level of life satisfaction are less prone to the detrimental effects of prolonged stress and competition demands, making it a crucial factor to consider in holistic athlete development (Glandorf et al., 2024).

In particular, SDT is a helpful theoretical framework for examining the possible motivational causes of athlete burnout. The included study suggests that burnout can cause a lack of motivation, however, it conflicts with other studies suggesting that amotivation can predict athlete burnout symptoms (Martinent et al., 2014). Because of the relatively short data collection period (2 months), there may not have been as much diversity in burnout in this study, which could have hidden increases in burnout among athletes who exhibit higher levels of amotivation. The interval between repeated measurements could also be the basis for the mixed results regarding the relationship between sleep problems and athlete burnout. Two of the three studies found that athlete burnout affects sleep, but sleep does not affect burnout symptoms. The main finding is that instead of being viewed as a result of symptoms of sleeplessness, burnout could be considered a cause. Athletes with clinically relevant burnout symptoms report significantly more insomnia symptoms, report more dysfunctional sleep-related cognitions, spend less time in bed during weekday nights, and report higher sleep-onset latency, both during weeknights and weekend nights (Gerber et al., 2018). One explanation could be that burnout is a symptom of stress, and stress is known to cause sleep issues. It can also be explained by the fact that athletes with high burnout levels tend to worry more about everything related to the training and competition process, which in turn can result in poor sleep quality (Li et al., 2018). The third study obtained opposite results, concluding that sleep disruptions predicted increases in sports devaluation (Glandorf et al., 2024). The difference in results is also explained by the fact that there could be other external factors that influence the results, for example, no information was collected on whether the participant uses any sleep medication or information on how the participant evaluates the quality of his sleep. Depression is another component that is discussed in the literature. The study included in this review demonstrated a unidirectional relationship between athlete burnout and depressive symptoms. Specifically, it was proven that exhaustion predicted increases in depressive symptoms (Glandorf et al., 2024). However, the results of other studies indicate that this relationship is bidirectional.

From The Commitment-based model perspective, factors that affect individuals’ perceptions of their involvement in sports would be included in this category. For instance, perfectionistic strivings appear to be a protective factor for athlete burnout, since commitment is based on attraction (“want to”) or entrapment (“have to”). Identity can influence this commitment. Identity leadership (encompassing identity prototypicality, advancement, entrepreneurship, and impresarioship) and team identification were positively related to teammates’ well-being and negatively to their burnout, however, coaches’ identity impresarioship was not significant. It was concluded that empowering athlete leaders and strengthening their identity leadership skills is an important way to unlock sports teams’ full potential (Fransen et al., 2022). Similarly, satisfaction and emotional connection are negatively associated with athlete burnout, nevertheless, a sense of belonging was positively associated with a reduced sense of accomplishment, and emotional connection with peers was positively linked to physical exhaustion (Shannon et al., 2022). The authors suggest that an overly strong sense of belonging to the community may undermine athletes’ autonomy, potentially leading to feelings of inefficacy in their sports performance and accomplishments. Another explanation relates to athletes’ sense of belonging within the intensive training center environment, which may diminish the perceived importance of accomplishments and increase motivation to train and perform out of a desire to avoid shame or guilt (Scotto di Luzio et al., 2020). In this regard, the relationship between coaches and athletes is crucial (Mellano et al., 2022). Coaches, in particular, should consider this while considering how to best deliver feedback to their athletes. They should attempt to use feedback that positively acknowledges their players’ accomplishments and minimize the frequency of punishment-based comments (Mellano et al., 2022).

### 4.2 Longitudinal insights into athlete burnout

The inclusion of only longitudinal studies strengthens the validity of this review by enabling insights into burnout’s progression over time. Unlike cross-sectional designs, longitudinal approaches allow for the examination of causal relationships and temporal changes, as evidenced by studies demonstrating significant increases in burnout dimensions such as sport devaluation during competitive seasons (Pires and Ugrinowitsch, 2021). The research procedure and the interval between repeated measurements also determine whether it will be possible to observe changes. The variability in measurement intervals across studies—ranging from 1 month to 1 year proposes—challenges in establishing optimal monitoring frequencies. Two research with a one-month interval found that neither the overall burnout nor any of the burnout dimensions changed significantly between waves. No athlete experienced a progressive increase in athlete burnout over time (Martinent et al., 2014, 2020). This result was explained by the fact that this interval is too short, as well as the fact that the data were collected towards the end of the season. So, not only the interval is important, but also the part of the season in which the data is collected. If the data is collected at the end of the season, it is possible that athlete burnout scores might have already attained their highest levels, leading to stabilization. Research suggests that a three-month interval, particularly when aligned with sports season phases, provides a balanced approach to capturing meaningful changes (Madigan et al., 2016a). Linking data collection to the phases of the sports season (e.g., beginning, middle, and end) ensures that the study considers season-specific factors that may influence athlete burnout. This approach helps to better understand how burnout symptoms increase or decrease depending on the stage of the season. It is most often observed that at the end of the season, the burnout of athletes becomes more pronounced compared to the beginning of the season (Kelecek and Koruc, 2022). A person-oriented approach is also an important aspect. In longitudinal studies, this appears as regular monitoring of burnout factors in order to be able to notice individual changes in the dynamics of burnout among athletes. It helps identify temporary changes and determine whether these changes are long-term or short-term.

Given the multidimensional nature of burnout, it is challenging to determine whether an athlete must exhibit high levels across all burnout dimensions or in just one dimension to be classified as experiencing burnout. Therefore, the Athlete Burnout Questionnaire provides an opportunity to analyze each of the dimensions separately and helps to draw correct conclusions. This is crucial when thinking about monitoring athlete burnout because studies have observed that each of these dimensions does not develop simultaneously, they vary over time, as well as influence each other. Since ABQ is a self-reported measurement, that can cause potential biases, including social desirability and recall inaccuracies. Future research should consider complementing self-reports with objective measures (e.g., physiological indicators of stress) to enhance reliability.

### 4.3 Athlete burnout prevention

Findings from the reviewed studies underscore the importance of early detection and personalized interventions. For example, the protective role of mindfulness and resilience in mitigating burnout symptoms suggests that integrating mental skills training into athletes’ routines could be a valuable preventative strategy (Amemiya and Sakairi, 2019; Sorkkila et al., 2019). Programs focusing on mindfulness, gratitude, and mental toughness have shown promise in enhancing coping resources and reducing stress, aligning with CAM’s emphasis on resource-demand balance.

From a coaching perspective, the detrimental impact of punitive feedback and the protective influence of autonomy-supportive behaviors (Mellano et al., 2022) highlight the need for coach education programs. Such programs should aim to develop coaches’ skills in delivering constructive feedback, fostering athlete autonomy, and building emotionally supportive relationships. Moreover, organizational interventions that address systemic stressors, such as high competition demands and inadequate recovery periods, are crucial in creating a supportive environment for athletes.

## 5 Limitations

This scoping review provides a summary of the psychological factors associated with athlete burnout that have been studied over the past 10 years. Despite the progress made, significant gaps remain in understanding athlete burnout. The limited examination of factors such as cultural differences, gender, and the interplay between personal and environmental influences warrants further investigation. For instance, studies have shown that female athletes may experience higher burnout rates in certain dimensions, such as emotional exhaustion and sport devaluation, compared to their male counterparts (Isoard-Gautheu et al., 2015). Exploring these variations can inform tailored interventions.

While all studies utilized repeated measurements, the intervals between these measurements varied, and different questionnaires were employed to assess athlete burnout. Due to the significant variability in longitudinal designs, it is not appropriate to draw definitive conclusions about the psychological factors that contribute most significantly to athlete burnout or its progression over time. Another limitation is that several psychological factors were examined in only one or a limited number of studies, making it difficult to draw definitive conclusions about their impact on athlete burnout. The limitations of the other included studies can also be added to the limitations of this study. All studies used only self-report questionnaires. That means that only a partial view of the phenomenon can be gained because there could be other factors that there was no way to control. Studies were conducted on a specific population, for example, adolescent handball players, or on the other hand were conducted on a wide range of athletes competing in various sports, which might limit the generalizability of the findings. Also, in some of the research there was a small number of participants and in others was an unbalanced number of athletes from individual and team sports.

Additionally, the high dropout rates observed in longitudinal studies highlight the need for strategies to improve participant retention. Incorporating digital tools for remote monitoring and gamification elements to enhance engagement could address this challenge. Lastly, qualitative research capturing athletes’ lived experiences of burnout would complement quantitative findings, providing a more holistic understanding of this complex phenomenon.

## 6 Conclusion

This scoping review of longitudinal studies on athlete burnout provides valuable insights into the complex and dynamic nature of this phenomenon. The review demonstrates that athlete burnout evolves in a nonlinear manner and can be understood either holistically through its total score or by examining its specific dimensions—emotional and physical exhaustion, reduced sense of accomplishment, and sport devaluation. These dimensions often develop independently, underscoring the multidimensional nature of burnout.

The analysis highlights the importance of consistent athlete burnout monitoring as the most effective means of capturing the dynamics of athlete burnout and the interplay between environmental and personal factors. The findings indicate that it is significant to choose a measurement tool suitable for the sample, which includes all dimensions of burnout since it is essential to analyze each of these dimensions separately to obtain the most accurate results. Additionally, determining an appropriate interval between repeated measurements is crucial. Such intervals should adopt a person-oriented approach to accurately capture the dynamics of athlete burnout. This involves considering the stage of the competitive season and how many months are between stages of the season (e.g., 3 months between the beginning and midpoint of the season). Moreover, it is also important to choose a long-term interval to be able to assess whether the existing burnout changes are only short-term or persist in the long-term. Accurate and systematic monitoring can play a pivotal role in mitigating the risk of burnout by enabling timely interventions.

From the results obtained, it was concluded that a range of factors contributes to the onset and progression of athlete burnout. Identifying risk factors (e.g., perfectionistic concerns, negative social interactions) and protective factors (e.g., social support, mental toughness) can be useful in developing interventions for athlete burnout. It is important to note that when developing interventions, it is essential to pay attention not only to the fact that they incorporate several syndromes but also to the fact that they depend on the time of year (considering the competition season). These findings hold significant practical implications for stakeholders in the sports domain, including coaches, athletes, and sports psychologists, by facilitating the adoption of proactive strategies for early recognition and effective intervention.

In conclusion, this review underscores the necessity for continued longitudinal research to deepen our understanding of athlete burnout and inform the development of targeted interventions. By fostering a supportive environment and addressing both individual and contextual risk factors, it is possible to reduce the prevalence of burnout and enhance athlete well-being and performance.

## Data Availability

The original contributions presented in the study are included in the article/Supplementary material, further inquiries can be directed to the corresponding author.

## Footnotes

^1^ https://osf.io/f4azd/
